# *Lactobacillus reuteri* NK33 and *Bifidobacterium adolescentis* NK98 Alleviate *Escherichia coli*-Induced Depression and Gut Dysbiosis in Mice

**DOI:** 10.4014/jmb.2002.02058

**Published:** 2020-04-29

**Authors:** Sang-Kap Han, Jeon-Kyung Kim, Min-Kyung Joo, Kyung-Eon Lee, Seung-Won Han, Dong-Hyun Kim

**Affiliations:** 1 Neurobiota Research Center, Department of Life and Nanopharmaceutical Sciences, College of Pharmacy, Kyung Hee University, Seoul 02447, Republic of Korea; 2 Navipharm Inc., Suwon 16209, Republic of Korea

**Keywords:** *Lactobacillus reuteri*, *Bifidobacterium adolescentis*, depression, gut microbiota

## Abstract

*Lactobacillus reuteri* NK33 (NK33) and *Bifidobacterium adolescentis* NK98 (NK98) alleviate immobilization stress-induced depression. To understand the gut microbiota-mediated mechanisms of NK33 and NK98 against depression, we examined their effects on *Escherichia coli* K1 (K1)-induced depression and gut dysbiosis in mice. NK33, NK98, and their mixtures (1:1, 4:1, and 9:1) mitigated K1-induced depression and colitis. NK33 and NK98 additively or synergistically increased BDNF^+^/NeuN^+^ cell population and suppressed NF-κB action in the hippocampus. They alleviated gut dysbiosis by reducing the Proteobacteria population and increasing the Clostridia population. These results suggest that NK33 and NK98 may alleviate depression and colitis by ameliorating gut dysbiosis.

Excessive exposure to stressors such as social defeat, immobilization, antibacterials, and pathogen infection stimulate the release of adrenal hormones noradrenaline, adrenaline, and glucocorticoids through the activation of the hypothalamic-pituitary-adrenal (HPA) axis and the secretion of proinflammatory cytokines IL-6 and TNF- α. This results in the occurrence of psychiatric disorders, colitis, and gut dysbiosis [1, 2]. Treatment with 2,4,6- trinitrobenzenesulfonic acid (TNBS), a colitis inducer, causes colitis, gut dysbiosis, and cognitive impairment in mice [3]. Oral gavage of ampicillin causes gut dysbiosis, colitis, and anxiety/depression in mice [4]. Infection with pathogens such as TNBS-induced *Escherichia coli* and ampicillin-induced *Klebsiella oxytoca* in the intestine of mice causes colitis with psychiatric disorders, including anxiety/depression and memory impairment, through the activation of the microbiota-gut-brain (MGB) axis [3-5]. However, *Lactobacillus mucosae* NK41 alleviates *E. coli* K1 (K1)-induced cognitive impairment, depression, and colitis in mice by the amelioration of gut dysbiosis [5].

Gut microbiota consist of > 1,000 bacterial species in humans and animals [6] and display a variety of physiological functions such as modulation of the host’s immune and nervous systems and defense against pathogen attacks [6, 7]. However, the induction of gut opportunistic *E. coli* K1 by immobilization stress (IS) causes colitis and psychiatric disorders [5, 8]. *Bifidobacterium adolescentis* IM38 mitigates anxiety in IS-treated mice [9]. *Lactobacillus reuteri* NK33 (NK33), *B. adolescentis* NK98 (NK98), and their (1:1) mixture were also reported to alleviate IS-induced anxiety/depression in mice [10]. However, at present, the gut microbiota-mediated inhibitory mechanism of probiotics NK33 and NK98 against anxiety/depression remains unclear.

Therefore, in the present study, we examined the effects of NK33, NK98, and their mixtures (1:1, 4:1, and 9:1) on K1-induced gut dysbiosis, colitis, and depression in mice.

Male C57BL/6 mice (5 weeks of age; 19-21 g in weight) were supplied by Koatech Inc. (Republic of Korea). The animals were maintained under controlled condition (temperature, 20 ± 2°C; humidity, 50 ± 10%; light-dark cycle, 12 h). The mice received standard laboratory chow with tap water ad libitum. All animal experiments were approved by the Institutional Animal Care and Use Committee of the University (IACC No. KUASP(SE)-19-152) and were performed according to the University Guide for Laboratory Animal Care and Usage.

NK33 and NK98 were cultured in MRS broth (BD, USA) for probiotics and K1 was cultured in the tryptic soy broth (BD). Cultured bacteria were centrifuged (5,000 *g,* 20 min, 4oC) and suspended in 1% maltose.

The mice with K1-induced depression were prepared following a previous report [8]. To evaluate the effects of NK33 and NK98 on the occurrence of depression, the mice were randomly divided into seven groups (CON, EC, EN33, EN98, EN1:1, EN4:1, and EN9:1), with five animals in each group. All groups except the CON group were orally gavaged with K1 (1 × 10^9^ CFU/mouse) daily for 5 days. The test agents (CON, 1% maltose; EC, 1% maltose; EN33, 1 × 10^9^ CFU/mouse/day of NK33; EN98, 1 × 10^9^ CFU/mouse/day of NK98; EN1:1, 1 × 10^9^ CFU/mouse/day of the NK33 and NK98 (1:1) mixture; EN4:1, 1 × 10^9^ CFU/mouse/ of the NK33 and NK98 (4:1) mixture; and EN9:1, 1 × 10^9^ CFU/mouse/ of the NK33 and NK98 (9:1) mixture) were orally gavaged once a day for 5 days from 24 h after the final K1 treatment.

Following previous reports [5, 8], depression-like behaviors were measured in the elevated plus maze (EPM) task, forced swimming test (FST), and tail suspension test (TST) 18 h after the final treatment with test agents. Corticosterone, TNF-α, and IL-6 levels were assayed by using enzyme-linked immunosorbent assay kits, as described in Supplement Methods. Immunohistochemistry was performed according to the method of Kim *et al.* [5].

Microbiota pyrosequencing was performed using Illumina iSeq 100 (USA), as previously reported [11]. Pyrosequencing reads were deposited in the NCBI’s short read archive under the accession number PRJNA603024. Experimental data were indicated as mean ± standard deviation (SD) and analyzed by Graph-Pad Prism 8 (GraphPad Software Inc., USA). The significance for data was analyzed by using one-way analysis of variance with Tukey's multiple comparison test (*p* < 0.05).

First, the effects of NK33 and NK98 on K1-induced anxiety/depression in mice were examined (Figs. 1A and B). Oral gavage of K1 significantly increased the immobility times in the TST and FST to 179% and 141% of the control group, respectively. However, NK33, NK98, and their mixtures (1:1, 4:1, and 9:1) inhibited the EC- induced depression-like behaviors: they decreased the immobility times in TST to 107%, 92%, 107%, 94%, and 104% of the control mice, respectively, and in FST to 92%, 89%, 96%, 94%, and 101% of the control mice, respectively. Exposure to K1 also increased IL-6 and TNF-α expression and NF-κB activation in the hippocampus and corticosterone and IL-6 levels in the blood (Figs. 1C- E). Oral administration of NK33, NK98, and their mixtures suppressed K1-induced IL-6 and TNF-α expression and NF-κB activation in the hippocampus (Figs. 1C- E), while the K1-suppressed BDNF^+^/NeuN^+^ cell population was increased in the hippocampal CA3 region (Fig. 1F). The treatments also reduced corticosterone and IL-6 in the blood (Figs. 1G and H). NK38 and NK98 additively or synergistically suppressed depression-like behaviors, IL-6 expression in the hippocampus, and corticosterone and IL-6 levels in the blood (Figs. 1A-C, G-H).

Oral gavage of K1 significantly induced colon shortening, myeloperoxidase activity, and TNF-α and IL-6 expression in the colon of mice (Figs. 2A-D). K1-induced myeloperoxidase activity and IL-6 expression were significantly suppressed by the treatment with NK33, NK98, or their mixtures (1:1, 4:1, and 9:1). NK38 and NK98 additively or synergistically suppressed these colitis markers.

Next, we examined whether K1 would cause gut dysbiosis, and whether probiotics NK33 and NK98 would alleviate K1-induced gut dysbiosis in mice (Fig. 3, Supplement Fig. S1, Supplement Tables S1, S2, S3). Treatment with NK33, NK98, and their mixtures increased K1-suppressed α-diversity based on OTU and Shannon indices and shifted β-diversity using the principal coordinate analysis (PCoA) based on Jensen-Shannon analysis. The bacterial community of mouse feces was significantly affected by K1 treatment. Treatment with NK33 or NK98 partially shifted the K1-dependent, altered gut microbiota composition. In particular, NK33 more potently shifted K1-altered gut microbiota composition to those of the control mice than did NK98. At the phylum level, exposure to K1 increased the Proteobacteria and Firmicutes populations as compared to those of the control mice. K1 treatment significantly increased Helicobacteriaceae populations, while the Prevotellaceae population was lower. However, NK33, NK98, and their mixtures suppressed the K1-increased Preoteobacteria population, while the Tenericutes population increased. Furthermore, NK33 increased Muribacteriaceae and PAC001068_g populations in K1-treated mice. NK98 increased PAC001770_s and KE159538_3 populations in K1-treated mice. Their mixtures (1:1, 4:1, and 9:1) increased Clostridia and Lachnospiraceae populations, PAC001120_s and *Helicobacter rodentium* group populations, and KE159538_g and KE159571_g populations, respectively.

Gut microbiota communicate to the brain through the HPA and MGB axes [2, 12]. Exposure to stressors causes psychiatric disorders such as anxiety and depression [2]. The prevalence of such disorders is significantly higher in patients with intestinal bowel disease than in healthy people [13]. A signature of intestinal bowel disease is gut dysbiosis marked by a decrease in obligate anaerobes such as *Faecalibacterium prausnitzii* and an increase in facultative anaerobes such as *E. coli* [14]. Gut dysbiosis causes psychiatric disorders including anxiety and depression with gut inflammation through the disturbance of the immune and nervous systems [15-17].

In the present study, we found that exposure of mice to K1 (bacterial infection) caused gut dysbiosis in these animals and increased their Proteobacteria populations, which is a common factor in human diseases [18]. The Bacteroidetes and Tenericutes populations were reduced. NK38, NK98 and their mixtures alleviated K1-induced gut dysbiosis, suppressed K1-induced Proteobacteria populations, and increased the K1-suppressed Tenericutes population. Furthermore, consistent with previous reports [5, 8], exposure to *E. coli* caused colitis and depression in mice. These results suggest that gut dysbiosis due to overgrowth of commensal *E. coli* in the gastrointestinal tract can induce depression and colitis through activation of the MGB axis. However, oral administration of NK33, NK98, and their mixtures were found to alleviate K1-induced anxiety/depression and colitis in mice. They also increased K1-suppressed BDNF^+^/NeuN^+^ cell population in the hippocampus. NK33 and NK98 additively or synergistically alleviated NF-κB activation and TNF-α and IL-6 expression in the hippocampus and IL-6 and corticosterone levels in the blood. Jang *et al*. reported that NK33 and NK98 alleviated immobilization stress- induced anxiety/depression in mice by regulating gut immune responses such as NF-κB activation and inducing brain-derived neurotropic factor (BDNF) expression [10]. *Lactobacillus johnsonii*, a commensal gut bacterium of mice, significantly suppresses stress-induced blood IL-6 and corticosterone levels, increases BDNF expression, and alleviates anxiety in mice [3, 4]. *Lactobacillus plantarum* suppresses anxiety-like behaviors associated with gut dysbiosis [19]. *Bifidobacterium breve* strain A1 alleviates cognitive impairment in mice by suppressing β-amyloid- induced inflammatory gene expression [20]. *L. plantarum* C29 improves cognitive function in 5XFAD mice and patients with mild cognitive decline by suppressing NF-κB activation and increasing BDNF expression [21, 22]. *L. mucosae* NK41 alleviates psychiatric disorders including cognitive impairment and depression in mice through the modulation of NF-κB activation and gut dysbiosis [5]. Antidepressant drugs, such as amitriptyline, also alleviate depression with colitis by suppressing the NF-κB signaling pathway [23]. These findings suggest that NK33 and NK98 can alleviate psychiatric disorders including anxiety/depression via the regulation of NF-κB- mediated BDNF expression. Furthermore, in the present study, NK33, NK98, and their mixtures were found to modulate K1-induced gut dysbiosis in mice by suppressing Helicobacteriaceae populations and inducing Clostridia populations, resulting in the attenuation of gut inflammation through the suppression of NF-κB activation. These results suggest that NK33 and NK98 can alleviate depression associated with gut inflammation through the modulation of gut microbiota composition.

Based on these findings, it can be concluded that NK33 and NK98 can additively or synergistically alleviate depression and colitis via the amelioration of gut dysbiosis.

## Supplemental Materials



Supplementary data for this paper are available on-line only at http://jmb.or.kr.

## Figures and Tables

**Fig. 1:**
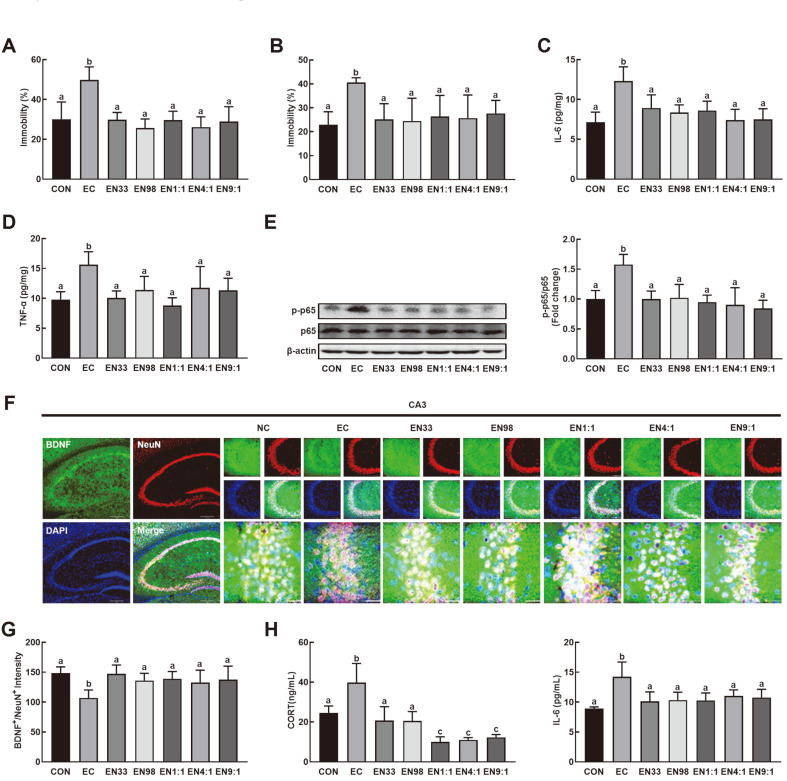
Oral gavage of *Lactobacillus reuteri* NK33 and/or *Bifidobacterium adolescentis* NK98 alleviated *Escherichia coli* K1-induced depression-like behaviors in mice. Effects on depression-like behaviors in the TST (**A**), and FST (**B**). Effects on IL-6 (**C**) and TNF-α expression (**D**), NF-κB activation (p-p65/p65, **E**), and BDNF^+^/NeuN^+^ cell populations (**F**) in the hippocampus. Effects on corticosterone (CORT, G) and IL-6 (**H**) levels in the blood. Mice were orally exposed to K1 and thereafter test agents (EC, vehicle [1% maltose]; EN33, 1 × 10^9^ CFU/mouse/day of NK33; EN98, 1 × 10^9^ CFU/mouse/day of NK98; EN1:1, 1 × 10^9^ CFU/mouse/day of the (1:1) mixture of NK33 and NK98]; EN4:1, 1 × 10^9^ CFU/ mouse/day of the (4:1) mixture of NK33 and NK98]; EN9:1, 1 × 10^9^ CFU/mouse/day of the (9:1) mixture of NK33 and NK98]) were orally gavaged daily for 5 days. CON group was treated with vehicle instead of test agents and K1. Blood corticosterone and IL-6 levels were assayed by ELISA kits. All data were expressed as mean ± SD (*n* = 5). Means with same letters are not significantly different (*p* < 0.05).

**Fig. 2:**
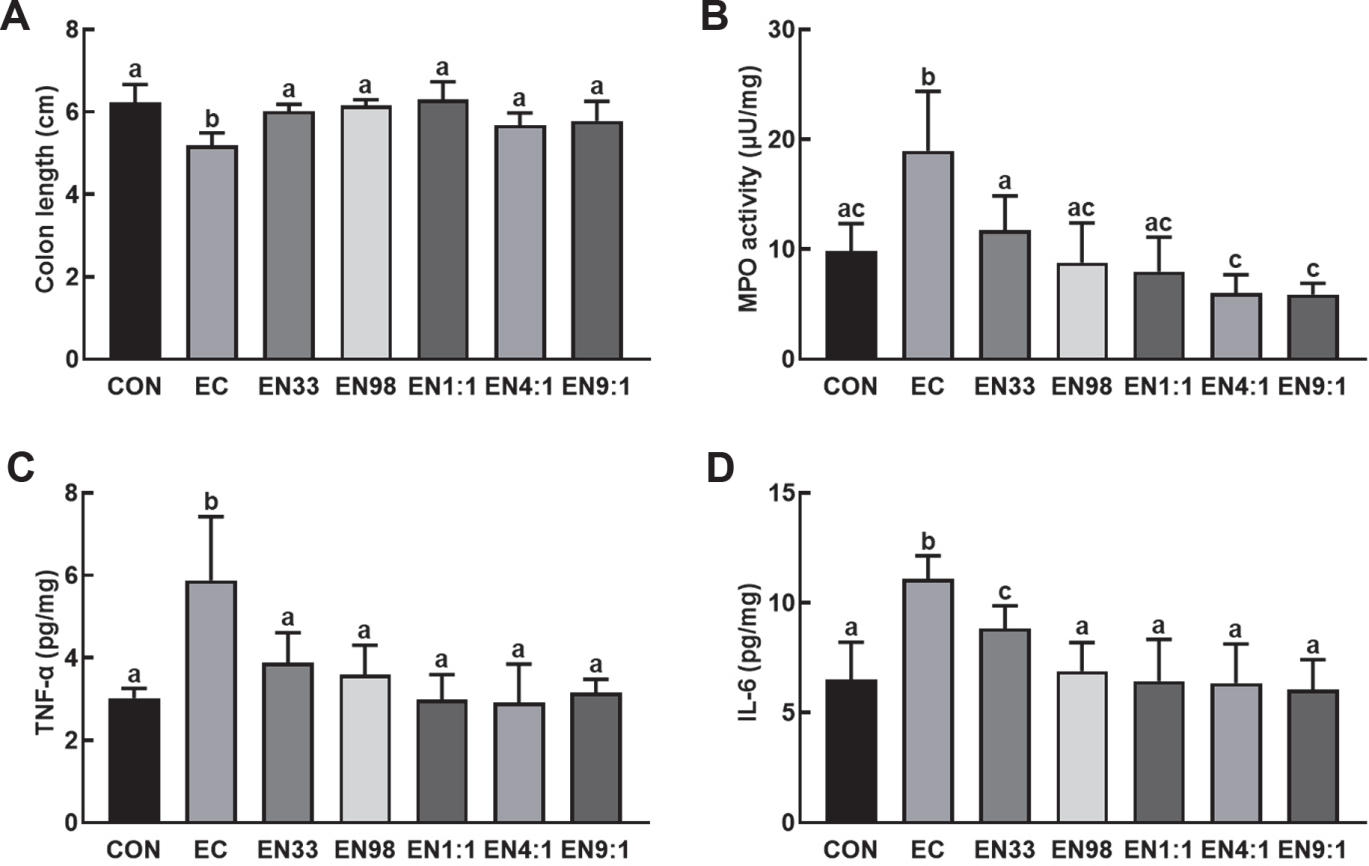
Oral gavage of *Lactobacillus reuteri* NK33 and/or *Bifidobacterium adolescentis* NK98 alleviated *Escherichia coli* K1-induced colitis in mice. Effects on colon length (**A**), myeloperoxidase (MPO) activity (**B**), and TNF- α (**C**) and IL-6 expression (**D**). Mice were treated with K1 and/or test agents, as described in Figure 1. All data were expressed as mean ± SD (*n* = 5). Means with same letters are not significantly different (*p* < 0.05).

**Fig. 3:**
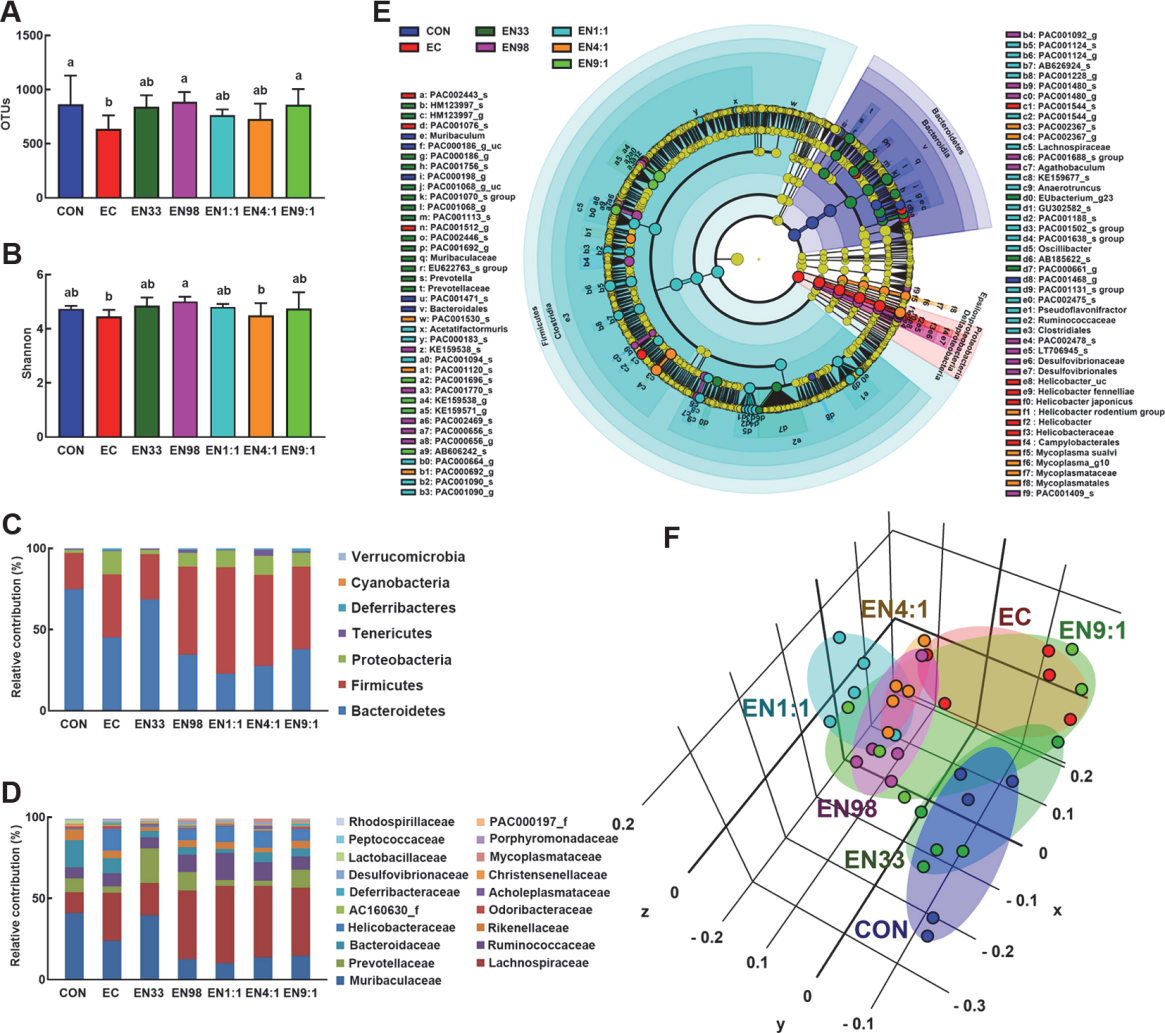
Oral gavage of *Lactobacillus reuteri* NK33 and/or *Bifidobacterium adolescentis* NK98 shifted *Escherichia coli* K1-induced gut microbiota alteration in mice. Effects on the composition of gut microbiota, analyzed by the pyrosequencing: OTUs (**A**) and Shannon’s diversity (**B**), principal coordinate analysis (PCoA) plot based on Jensen-Shannon analysis (**C**), phylum (**D**), family (**E**), and cladogram (**F**) generated by LEfSE indicating significant differences in gut microbial abundances among CON (blue), EC (red), EN33 (purple), EN98 (green), EN1:1 (blue-green), EN4:1 (orange), and EN9:1 (chartreuse) groups. The threshold logarithmic score set at 3.5 and ranked. Yellow nodes represent species with no significant difference. Mice were treated with *Escherichia coli* K1 and test agents, as described in Figure 1. All data were expressed as mean ± SD (*n* = 5). Means with same letters are not significantly different (*p* < 0.05).

## References

[1] El Aidy S, Dinan TG, Cryan JF (2014). Immune modulation of the brain-gut-microbe axis. Front. Microbiol..

[2] Yarandi SS, Peterson DA, Treisman GJ, Moran TH, Pasricha PJ (2016). Modulatory effects of gut microbiota on the central nervous system: How gut could play a role in neuropsychiatric health and diseases. J. Neurogastroenterol. Motil..

[3] Jang SE, Lim SM, Jeong JJ, Jang HM, Lee HJ, Han MJ, et al (2018). Gastrointestinal inflammation by gut microbiota disturbance induces memory impairment in mice. Mucosal Immunol..

[4] Jang HM, Lee HJ, Jang SE, Han MJ, Kim DH (2018). Evidence for interplay among antibacterial-induced gut microbiota disturbance, neuro-inflammation, and anxiety in mice. Mucosal Immunol..

[5] Kim JK, Lee KE, Lee SA, Jang HM, Kim DH (2020). Interplay between human gut bacteria *Escherichia coli* and *Lactobacillus mucosae* in the occurrence of neuropsychiatric disorders in mice. Front. Immunol..

[6] Thursby E, Juge N (2017). Introduction to the human gut microbiota. Biochem. J..

[7] Lozupone CA, Stombaugh JI, Gordon JI, Jansson JK, Knight R (2012). Diversity, stability and resilience of the human gut microbiota. Nature.

[8] Jang HM, Lee KE, Lee HJ, Kim DH (2018). Immobilization stress-induced *Escherichia coli* causes anxiety by inducing NF-κB activation through gut microbiota disturbance. Sci. Rep..

[9] Jang HM, Jang SE, Han MJ, Kim DH (2018). Anxiolytic-like effect of *Bifidobacterium adolescentis* IM38 in mice with or without immobilisation stress. Benef. Microbes..

[10] Jang HM, Lee KE, Kim DH (2019). The Preventive and curative effects of *Lactobacillus reuteri* NK33 and *Bifidobacterium adolescentis* NK98 on immobilization stress-induced anxiety/depression and colitis in mice. Nutrients.

[11] Jang HM, Han SK, Kim JK, Oh SJ, Jang HB, Kim DH (2019). *Lactobacillus sakei* alleviates high-fat-diet-induced obesity and anxiety in mice by inducing AMPK activation and SIRT1 expression and inhibiting gut microbiota-mediated NF-κB activation. Mol. Nutr. Food Res..

[12] Zhu X, Han Y, Du J, Liu R, Jin K, Yi W (2017). Microbiota-gut-brain axis and the central nervous system. Oncotarget.

[13] Graff LA, Walker JR, Bernstein CN (2009). Depression and anxiety in inflammatory bowel disease: a review of comorbidity and management. Inflamm. Bowel Dis..

[14] Bernstein CN (2016). Psychological stress and depression: Risk factors for IBD?. Dig. Dis..

[15] Van Ameringen M, Turna J, Patterson B, Pipe A, Mao RQ, Anglin R, et al (2019). The gut microbiome in psychiatry: A primer for clinicians. Depress. Anxiety.

[16] Rogers GB, Keating DJ, Young RL, Wong ML, Licinio J, Wesselingh S (2016). From gut dysbiosis to altered brain function and mental illness: mechanisms and pathways. Mol. Psychiatry.

[17] Galland L (2014). The gut microbiome and the brain. J. Med. Food.

[18] Rizzatti G, Lopetuso LR, Gibiino G, Binda C, Gasbarrini A (2017). Proteobacteria: A common factor in human diseases. Biomed. Res. Int..

[19] Davis DJ, Doerr HM, Grzelak AK, Busi SB, Jasarevic E, Ericsson AC, et al (2016). *Lactobacillus plantarum* attenuates anxiety-related behavior and protects against stress-induced dysbiosis in adult zebrafish. Sci. Rep..

[20] Kobayashi Y, Sugahara H, Shimada K, Mitsuyama E, Kuhara T, Yasuoka A, et al (2017). Therapeutic potential of *Bifidobacterium breve* strain A1 for preventing cognitive impairment in Alzheimer's disease. Sci. Rep..

[21] Lee HJ, Hwang YH, Kim DH (2018). *Lactobacillus plantarum* C29-Fermented soybean (DW2009) alleviates memory impairment in 5XFAD transgenic mice by regulating microglia activation and gut microbiota composition. Mol. Nutr. Food Res..

[22] Hwang YH, Park S, Paik JW, Chae SW, Kim DH, Jeong DG, et al (2019). Efficacy and safety of *Lactobacillus plantarum* C29-fermented soybean (DW2009) in individuals with mild cognitive impairment: A 12-week, multi-center, randomized, double-blind, placebo-controlled clinical trial. Nutrients.

[23] Fattahian E, Hajhashemi V, Rabbani M, Minaiyan M, Mahzouni P (2016). Anti-inflammatory effect of amitriptyline on ulcerative colitis in normal and reserpine-induced depressed rats. Iran J. Pharm. Res..

