# Supplementary material for: Lactobacillus reuteri NK33 and Bifidobacterium adolescentis NK98 Alleviate Escherichia coli-Induced Depression and Gut Dysbiosis in Mice
Source: J Microbiol Biotechnol. 2020 Apr 29;30(8):1222–6. doi: 10.4014/jmb.2002.02058 (PMC9728327; doi:10.4014/jmb.2002.02058)
Supplement: Supplementary file 1 [file JMB-30-8-1222-supple.pdf]

J. Microbiol. Biotechnol.
https://doi.org/10.4014/jmb.2002.02058

[Supplement]

[illegible]acillus reuteri NK33 and *Bifidobacterium adolescentis* NK98 alleviate *Escherichia coli*-induced depression in mice by the amelioration of colitis and gut dysbiosis

[Methods]

Behavioral tasks

The tail suspension (TS) task was measured according to the method of Jang et al. [1]. Mice were suspended on the edge of a table 30 cm above the floor by taping 1 cm from the tail tip. Immobility time was measured for 5 min. Mice were judged to be immobile, when they did not move and hanged passively.

The forced swimming (FS) task was performed in a round transparent plastic jar (20 × 40 cm3) containing fresh water (25°C) to a height of 25 cm [1]. Immobility time was measured during 5 min. Mice were judged to be immobile, when they remained floating in the water without struggling.

ELISA assay

The bloods collected from carotid artery and hippocampal and colon tissue homogentates were centrifuged at 3000 g, 4°C for 5 min and levels of corticosterone and cytokines TNF-α and IL-6 in the resulting supernatants of tissue homogenates and corticosterone in the plasma (the supernatant of blood) were assessed using ELISA kits [1].

Microbiota pyrosequencing

Genomic DNA was extracted from the fresh stools of five mice (not trans-cardiacally

perfused with 4% paraformaldehyde for brain and colon tissue sections) using a commercial DNA isolation kit (QIAamp DNA stool mini kit), as previously reported [1]. Briefly, genomic DNA was extracted from the fresh stools of mice using a commercial DNA isolation kit (QIAamp DNA stool mini kit). Amplification of the genomic DNA was performed using barcoded primers, which targeted the V4 region of the bacterial 16S rRNA gene, described in Supplement. Sequencing for each amplicon was performed using Illumina iSeq 100 (San Diego, CA). Predictive functional genes were analyzed using the phylogenetic investigation of communities by reconstruction of unobserved states (PICRUSt) [2]. Linear discriminant analysis (LDA) analysis and cladograms were developed on family level data using LDA effect size (LefSe) on Galaxy platform (https://huttenhower.sph.harvard.edu/galaxy/). Pyrosequencing reads have been deposited in the NCBI's short read archive under accession number PRJNA603024.

[Figure]

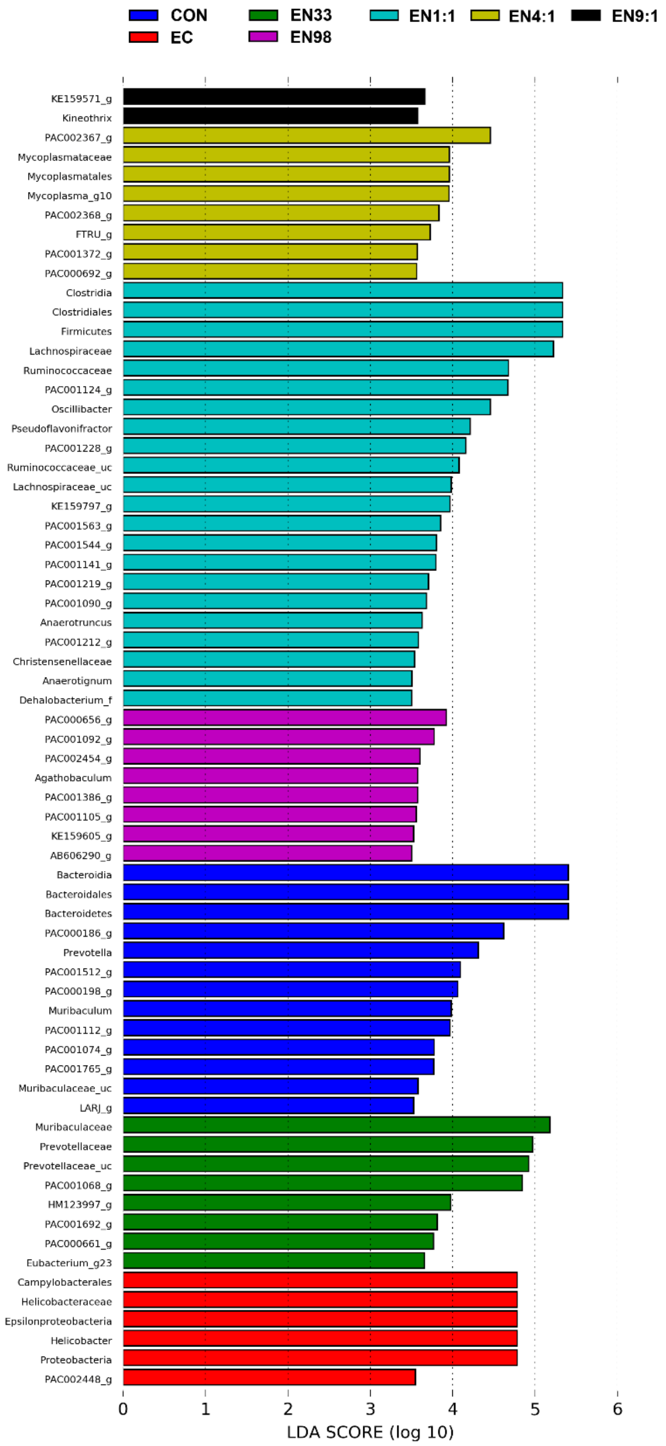

Figure S1. Effects of NK33, NK98, and their mixtures on gut microbiota composition in mice with *Escherichia coli* K1-induced anxiety/depression. The described strains were analyzed to

the Linear Discriminant Analysis (LDA) along with effect size measurement (LEfSE) in Galaxy (http://huttenhower.sph.harvard.edu/galaxy/). It was used to discriminate significant differentially strains at each taxon level. The threshold logarithmic score set at 3.5 and ranked. Bacterial strains were described based on 16SrRNA sequencing data. CON (blue), EC (red), EN33 (purple), EN98 (green), EN1:1 (blue-green), EN4:1 (orange), and EN9:1 (chartreuse) indicate groups treated with vehicle alone treated control, *Escherichia coli* K1 alone, NK38 with *Escherichia coli* K1, NK98 with *Escherichia coli* K1, NK33/NK98 (1:1) mix with *Escherichia coli* K1, NK33/NK98 (4:1) mix with *Escherichia coli* K1, and NK33/NK98 (9:1) mix with *Escherichia coli* K1, respectively.

Table S3. Effects of NK33 and NK98 on the gut microbiota composition at the phylum level in mice with EC-induced depression

Taxon Name	AVE							STD						
	CON	EC	EN33	EN98	EN1:1	EN4:1	EN9:1	CON	EC	EN33	EN98	EN1:1	EN4:1	EN9:1
Actinobacteria	0.01	0.01	0.02	0.01	0.00	0.01	0.01	0.02	0.01	0.02	0.01	0.01	0.02	0.01
Bacteroidetes	75.02	45.39[#]	68.84*	34.64	22.86	27.64	38.14	4.58	19.79	9.90	11.33	11.17	7.80	10.62
Cyanobacteria	0.11	0.07	0.04	0.16	0.08	0.17	0.12	0.15	0.04	0.03	0.12	0.07	0.19	0.15
Deferribacteres	0.02	0.89	0.08	0.73	0.57	0.46	1.53	0.02	1.42	0.05	0.69	0.80	0.88	1.32
Firmicutes	22.46	38.73[#]	27.86	54.26	65.55*	56.00	50.82	5.04	13.70	9.86	9.03	7.55	10.78	9.64
Proteobacteria	1.62	14.42[#]	2.45*	8.51	10.39	12.01	8.23	1.47	5.33	0.71	5.44	4.60	5.10	2.94
Saccharibacteria_TM7	0.00	0.00	0.00	0.00	0.00	0.01	0.00	0.00	0.00	0.01	0.00	0.00	0.01	0.01
Tenericutes	0.75	0.37	0.67	1.69	0.55	3.62	1.14	1.12	0.52	0.45	1.86	0.60	7.13	1.18
Verrucomicrobia	0.01	0.11[#]	0.02	0.00*	0.00*	0.08	0.01*	0.01	0.09	0.02	0.00	0.00	0.08	0.01

Mice were orally exposed to K1 and thereafter test agents (EC, vehicle [1% maltose]; EN33, 1×10^9 CFU/mouse/day of NK33; EN98, 1×10^9 CFU/mouse/day of NK98; EN1:1, 1×10^9 CFU/mouse/day of the (1:1) mixture of NK33 and NK98]; EN4:1, 1×10^9 CFU/mouse/day of the (4:1) mixture of NK33 and NK98]; EN9:1, 1×10^9 CFU/mouse/day of the (9:1) mixture of NK33 and NK98]) were orally gavaged daily for 5 days. CON group was treated with vehicle instead of test agents and K1. $^{\#}p<0.05$ vs. CON group. $^{*}p<0.05$ vs. EC group.

Table S3. Effect of NK33 and NK98 on the gut microbiota composition at the family level in mice with EC-induced depression

Taxon Name	AVE							STD						
	CON	EC	EN33	EN98	EN1:1	EN4:1	EN9:1	CON	EC	EN33	EN98	EN1:1	EN4:1	EN9:1
AC160630_f	0.62	0.91	0.22	0.26	0.31	0.58	0.22	0.95	1.13	0.08	0.30	0.14	0.65	0.09
Acholeplasmataceae	0.01	0.30	0.07	0.24	0.35	1.94	0.17	0.01	0.46	0.07	0.45	0.63	4.06	0.35
Bacteroidaceae	16.62	9.08	3.94	4.51	2.63	6.05	5.19	14.80	8.63	1.74	1.41	2.17	5.62	3.87
Christensenellaceae	0.42	0.56	0.21*	0.66	0.82	0.58	0.53	0.12	0.30	0.07	0.31	0.36	0.35	0.40
Deferribacteraceae	0.02	0.89	0.08	0.73	0.57	0.46	1.53	0.02	1.42	0.05	0.69	0.80	0.88	1.32

Desulfovibrionaceae	0.18	0.70#	0.72	1.31	1.04	1.30	0.66	0.24	0.22	0.17	0.86	0.27	0.75	0.15
Helicobacteraceae	0.88	13.47#	1.43*	7.08	9.28	10.47	7.47	0.90	5.29	0.84	5.03	4.61	4.91	2.93
Lachnospiraceae	12.73	29.52#	19.76	42.23	47.33*	43.56	41.54	2.37	11.77	9.18	8.89	4.20	10.31	9.59
Lactobacillaceae	2.14	0.38	0.71	0.24	0.35	0.28	0.44	3.60	0.21	0.36	0.10	0.26	0.24	0.42
Muribaculaceae	41.40	24.48#	39.92*	12.97*	10.57*	14.32	15.42	5.36	11.56	7.39	3.23	5.87	3.85	5.93
Mycoplasmataceae	0.52	0.00	0.34	1.24	0.00	1.51	0.83	1.14	0.01	0.46	1.83	0.01	3.11	0.92
Odoribacteraceae	0.80	1.56	0.82	0.72	0.86	0.36*	1.55	0.95	0.91	1.52	1.17	0.58	0.40	1.75
PAC000197_f	0.15	0.05	0.21	0.17	0.14	0.12	0.10	0.11	0.05	0.30	0.12	0.18	0.18	0.10
Peptococcaceae	0.34	0.05	0.02	0.06	0.06	0.04	0.02	0.53	0.03	0.02	0.03	0.03	0.02	0.02
Porphyromonadaceae	0.44	0.51	0.44	0.46	0.50	0.34	0.31	0.31	0.36	0.22	0.10	0.36	0.12	0.15
Prevotellaceae	8.48	3.92	21.07*	11.36*	3.81	3.18	11.12	8.11	2.56	6.37	5.41	3.16	2.10	8.61
Rhodospirillaceae	0.18	0.22	0.07	0.04	0.02*	0.01*	0.03	0.20	0.18	0.03	0.04	0.04	0.01	0.06
Rikenellaceae	6.64	4.93	2.39	4.34	4.18	2.79	4.29	5.31	3.65	1.46	5.08	1.88	0.82	1.44
Ruminococcaceae	6.48	7.96	6.66	10.61	16.54*	11.11*	7.92	1.22	2.07	1.82	2.71	6.05	1.80	3.78

Mice were orally exposed to K1 and thereafter test agents (EC, vehicle [1% maltose]; EN33, 1 × 10^9 CFU/mouse/day of NK33; EN98, 1 × 10^9 CFU/mouse/day of NK98; EN1:1, 1 × 10^9 CFU/mouse/day of the (1:1) mixture of NK33 and NK98]; EN4:1, 1 × 10^9 CFU/mouse/day of the (4:1) mixture of NK33 and NK98]; EN9:1, 1 × 10^9 CFU/mouse/day of the (9:1) mixture of NK33 and NK98]) were orally gavaged daily for 5 days. CON group was treated with vehicle instead of test agents and K1. $^{\#}p<0.05$ vs. CON group. $^{*}p<0.05$ vs. EC group.

Table S3. Effect of NK33 and NK98 on the gut microbiota composition at the genus level in mice with EC-induced depression

Taxon Name	AVE							STD						
	CON	EC	EN33	EN98	EN1:1	EN4:1	EN9:1	CON	EC	EN33	EN98	EN1:1	EN4:1	EN9:1
Acholeplasma_g2	0.01	0.30	0.02	0.23	0.31	1.93	0.16	0.01	0.46	0.05	0.45	0.65	4.06	0.35
Alistipes	6.31	4.64	1.74	3.87	3.57	2.45	3.57	5.13	3.64	1.22	4.84	1.78	0.83	1.56
Alloprevotella	0.12	0.34	0.02	0.02	0.20	0.01	1.02	0.25	0.52	0.04	0.04	0.30	0.00	2.27
Bacteroides	16.05	9.07	3.94	4.51	2.62	6.04	5.18	14.03	8.62	1.74	1.42	2.16	5.60	3.85
Helicobacter	0.88	13.44#	1.43*	7.06	9.26	10.43	7.46	0.89	5.28	0.84	5.01	4.59	4.89	2.91
KE159538_g	1.13	12.76#	5.61	9.95	4.30	11.12	16.61	0.67	10.53	6.93	3.09	1.64	13.65	17.02
LLKB_g	0.25	2.39	0.69	2.42	2.52	0.99	3.69	0.23	3.43	0.93	1.41	0.64	0.31	3.41
Lachnospiraceae_uc	0.12	0.43#	0.20	0.77	1.91	1.26	1.04*	0.06	0.23	0.20	0.35	1.55	1.39	0.48
Lactobacillus	2.13	0.38	0.71	0.24	0.35	0.28	0.44	3.59	0.21	0.36	0.10	0.26	0.24	0.42
Oscillibacter	1.80	2.90	1.09	3.72	6.97*	4.46	1.87	1.14	1.73	0.47	1.31	3.45	0.78	0.99
PAC000186_g	11.63	4.46#	11.05*	3.67	3.66	4.77	3.79	1.70	2.53	3.29	1.15	2.22	1.60	1.57
PAC000198_g	2.47	0.99	1.64	0.84	0.48	0.63	0.70	1.63	0.68	0.60	0.47	0.18	0.26	0.47
PAC000664_g	1.36	3.14	1.82	4.06	6.36	2.98	4.57	0.38	1.77	0.91	1.38	4.42	1.87	2.38
PAC001063_g	7.43	1.11	0.09*	0.13	0.11	0.17	0.14	10.47	0.99	0.05	0.14	0.07	0.17	0.17
PAC001066_g	2.25	0.60	1.03	0.31	0.35	0.52	0.50	2.02	0.32	1.10	0.18	0.55	0.37	0.25
PAC001068_g	7.49	7.63	16.57*	3.24*	2.84*	4.45	5.75	6.72	3.96	4.99	0.77	1.76	1.63	2.00
PAC001091_g	2.22	2.50	1.98	1.49	0.99*	2.82	0.79*	1.38	1.28	2.68	2.08	0.51	3.26	0.32
PAC001124_g	0.09	0.00	1.32	5.19	8.48*	5.03*	2.58	0.11	0.01	2.28	5.62	6.79	3.99	3.49
PAC001228_g	1.09	0.77	0.44	1.67	3.19*	1.52	0.98	0.90	0.97	0.45	1.53	1.49	0.61	0.96
PAC001512_g	1.85	2.01	1.03	0.81	0.18*	0.32*	0.73	2.34	1.33	0.36	0.43	0.19	0.17	0.70
PAC002367_g	0.87	0.06	0.63	1.71	3.59	5.76*	0.78	1.23	0.08	1.34	2.86	4.41	3.27	1.06
Prevotellaceae_uc	4.65	0.48	16.43*	9.80*	3.19	2.13	7.05	5.80	0.63	8.00	5.20	3.31	1.88	7.64
Pseudoflavonifractor	1.58	3.15#	1.87	3.47	5.13*	3.90	3.18	0.45	1.00	0.80	1.27	1.50	1.07	1.26

Mice were orally exposed to K1 and thereafter test agents (EC, vehicle [1% maltose]; EN33, 1 × 10^9 CFU/mouse/day of NK33; EN98, 1 × 10^9 CFU/mouse/day of NK98; EN1:1, 1 × 10^9 CFU/mouse/day of the (1:1) mixture of NK33 and NK98]; EN4:1, 1 × 10^9 CFU/mouse/day of the (4:1) mixture of NK33 and NK98]; EN9:1, 1 × 10^9 CFU/mouse/day of the (9:1) mixture of NK33 and NK98]) were orally gavaged daily for 5 days. CON group was treated with vehicle instead of test agents and K1. $^{\#}p<0.05$ vs. CON group. $^{*}p<0.05$ vs. EC group.

References

[1] Jang HM, Lee KE, Lee HJ, Kim DH. 2018. Immobilization stress-induced Escherichia coli causes anxiety by inducing NF-κB activation through gut microbiota disturbance. *Sci. Rep.* 8:13897.

[2] Langille MG, Zaneveld J, Caporaso JG, McDonald D, Knights D, Reyes JA, Clemente JC, Burkepile DE, Vega Thurber RL, Knight R, Beiko RG, Huttenhower C. 2013. Predictive functional profiling of microbial communities using 16S rRNA marker gene sequences. *Nat. Biotechnol*. 31, 814-821.
